# *Saccharomyces cerevisiae* ER membrane protein complex subunit 4 (*EMC4*) plays a crucial role in eIF2B-mediated translation regulation and survival under stress conditions

**DOI:** 10.1186/s43141-020-00029-7

**Published:** 2020-06-01

**Authors:** Sonum Sharma, Anuradha Sourirajan, David J. Baumler, Kamal Dev

**Affiliations:** 1 Faculty of Applied Sciences and Biotechnology, Shoolini University, Solan, Himachal Pradesh 173212 India; 2 Department of Food Science and Nutrition, University of Minnesota-Twin Cities, St. Paul, MN USA; 3 Microbial and Plant Genomic Institute, University of Minnesota-Twin Cities, St. Paul, MN USA; 4 Biotechnology Institute, University of Minnesota-Twin Cities, St. Paul, MN USA

**Keywords:** *S. cerevisiae*, eIF2B, VWM, Suppression, Identification, Emc4p, General Control Derepressed (GCD), General Control Nonderepressible (GCN)

## Abstract

**Background:**

Eukaryotic initiation factor 2B (eIF2B) initiates and regulates translation initiation in eukaryotes. eIF2B gene mutations cause leukoencephalopathy called vanishing white matter disease (VWM) in humans and slow growth (Slg^−^) and general control derepression (Gcd^−^) phenotypes in *Saccharomyces cerevisiae.*

**Results:**

To suppress eIF2B mutations, *S. cerevisiae* genomic DNA library was constructed in high-copy vector (YEp24) and transformed into eIF2B mutant *S. cerevisiae* strains. The library was screened for wild-type genes rescuing *S. cerevisiae* (Slg^−^) and (Gcd^−^) phenotypes. A genomic clone, Suppressor-I (Sup-I), rescued *S. cerevisiae* Slg^−^ and Gcd^−^ phenotypes (*gcd7-201 gcn2∆*). The YEp24/Sup-I construct contained truncated *TAN1*, full length EMC4, full length YGL230C, and truncated SAP4 genes. Full length EMC4 (chaperone protein) gene was sub-cloned into pEG (KG) yeast expression vector and overexpressed in *gcd7-201 gcn2∆* strain which suppressed the Slg^−^ and Gcd^−^ phenotype. A GST-Emc4 fusion protein of 47 kDa was detected by western blotting using α-GST antibodies. Suppression was specific to *gcd7-201 gcn2∆* mutation in eIF2Bβ and *Gcd1-502 gcn2∆* in eIF2Bγ subunit. Emc4p overexpression also protected the wild type and mutant (*gcd7-201 gcn2∆*, *GCD7 gcn2∆*, and *GCD7 GCN2∆*) strains from H_2_O_2_, ethanol, and caffeine stress.

**Conclusions:**

Our results suggest that Emc4p is involved in eIF2B-mediated translational regulation under stress and could provide an amenable tool to understand the eIF2B-mediated defects.

## Background

Eukaryotic initiation factor 2B (eIF2B) a heterodecameric complex of five non-identical protein subunits (α–ε) initiates/regulates translation [1]. α, β, and δ subunits of eF2B constitute regulatory sub complex, while the γ and ε subunits form catalytic sub complex [2]. eIF2B initiates translation by catalyzing the GDP-GTP exchange on its substrate, eukaryotic initiation factor 2 (eIF2). Under stress, eIF2B tightly binds to the phosphorylated eIF2 [2–4] which reduces eIF2B activity, and a transcription-activating factor *GCN4* in *S. cerevisiae* and ATF4 in humans are translated [5, 6] inducing various stress response genes [7]. Mutations in eIF2B subunits cause a neurodegenerative disease, called VWM (leukoencephalopathy with vanishing white matter) [8–10]. In VWM patients, eIF2B GEF activities are generally lower than normal [11] and is insensitive to eIF2 (loss of eIF2B-eIF2 interaction) [12–14]. Low eIF2B activity induces *GCN4/*ATF4 even in absence of eIF2 phosphorylation [15–19] and induces stress-like conditions. Neurological disorder further provokes additional stress and white matter deterioration.

Regulatory subunits of eIF2B are important for eI2B-eIF2 interaction under normal and stress conditions. Archeal eIF2B interacts with eukaryotic eIF2α and eIF2Bα indicating the importance of regulatory subunits [20]. eIF2Bβ subunit binds eIF2 which is important for eIF2-eIF2B interaction and translation regulation [21]. During integrated stress response, mutations in eIF2Bβ subunit suppress translation and cause delay in the recovery [22]. Identifying extragenic suppressors, modulators (proteins/chemicals) of mutated eIF2B regulatory subunits, may be useful in curing VWM disease. The chemical modulators, activating either GCN4 or suppressing eIF2B mutations, have been previously identified [23]. The goal of our study was to identify the *S. cerevisiae* protein that interacts with mutated eIF2B subunit and suppresses the mutation.

eIF2B mutant *S. cerevisiae* strains with deletion of protein kinase Gcn2p (phosphorylates eIF2α) gene give general control derepression phenotype (Gcd^−^ phenotype) and slow growth (Slg^−^) phenotype*.* In Gcd^−^ phenotype, *GCN4* is activated even in absence of eIF2α phosphorylation. The qualitative measurement of eIF2B activity and *GCN4* activation in *gcn2 Δ* strains can be measured in vivo on 3-amino triazole (3-AT) plates. 3-Amino triazole (3-AT) is a histidine analog and causes amino acid (histidine) starvation in *S. cerevisiae*-activating Gcn2p kinase and Gcn4p expression. If there are mutations in eIF2B subunit genes in gcn2∆ strains, the GEF activity of eIF2B is reduced. This reduction in eIF2B GEF activity helps in the growth of *S. cerevisiae* strains on medium containing 3-AT. This assay is used for indirect expression of Gcn4p.

In the present study, overexpression of a wild-type *S. cerevisiae* chaperone protein ER transmembrane complex 4 (Emc4p) rescued both the Slg^−^ and Gcd^−^ phenotypes of *S. cerevisiae* strains containing mutations either in β (*gcd7-201*) or γ (*gcd1-502*) subunits. Here we observed that Emc4p overexpression confers resistant to the H_2_O_2_, ethanol, and caffeine stress, in mutant or wild-type cells. We proposed a model that Emc4p by its chaperone activity folds and stabilize the destabilized and unfolded eIF2Bβ and eIF2Bγ subunits. However, it is unclear why Emc4p cannot suppress the mutations in other subunits of eIF2B. But this clearly suggests that interaction of both the subunits eIF2Bβ and eIF2γ with each other is critical for eIF2B activity, and mutations in any of these subunits can cause VWM disease.

## Methods

All the chemicals and reagents were of molecular biology grade procured from Thermo scientific, Himedia Labs, India; MP Biomedicals, USA; Fermentas Inc. USA; and Bio-Rad. USA.

### *S. cerevisiae* strains and plasmids

*S. cerevisiae* strains employed in this study (Table S1) were cultured on YPD agar or liquid medium. *S. cerevisiae* transformants were selected on synthetic complete (SC) medium lacking uracil and supplemented with glucose/galactose/raffinose. *S. cerevisiae* strains were incubated at 30 °C. *E. coli* strain DH5α was used for *S. cerevisiae* genomic DNA library construction and plasmid isolation.

YEp24 (high copy shuttle vector) and pEG(KG) (yeast expression vector) were used for cloning and expression of *S. cerevisiae* genes respectively. Nutrient broth (NB, Himedia Labs, Mumbai) with 100 μg/ml ampicillin was used to culture the *E. coli* strain DH5α harboring YEp24 or pEG(KG) at 37 °C. Plasmid DNA of YEp24 and pEG(KG) were isolated and used in transformations of yeast strains [24, 25].

### Construction of *S. cerevisiae* genomic DNA library and transformation into eIF2B mutant *S. cerevisiae* strains

Genomic DNA from *S. cerevisiae* strain H4 (Table S1) was isolated and partially digested with *Sau3AI* enzyme [24]. Fifty nanograms of partially digested and gel purified (gel purification kit Thermo-scientific) genomic DNA was ligated with 20 μg of YEp24 vector at *BamHI* site using T4 DNA ligase [26]. After ligation at 16 °C for 16 h, *E. coli* strain DH5α was transformed with the ligation mix by heat shock method [24]. The transformation mix was plated on NA medium containing ampicillin (100 μg/ml). Transformations were selected against ampicillin resistance on NA medium containing ampicillin and were pooled into three groups named as pool-I, pool-II, and pool-III.

Plasmid DNA isolation from three pools indicating ~ 13,575 cfu (colony-forming units) of transformants of DH5α was done [24]. Plasmids isolated from all three pools or vector (YEp24) alone were transformed into *S. cerevisiae* eIF2B mutant strains (Figure S1). The wild-type strains were transformed with YEp24 vector alone using LiAc method [25]. The nomenclature used for various *S. cerevisiae* strains used in this study is given in (Table S2). Transformation mix was plated on synthetic complete (SC) medium containing 2% glucose lacking uracil. SC mixture lacking uracil was used as a dropout supplement to select transformants containing uracil-based plasmid. eIF2B mutant *S. cerevisiae* transformants with normal colony size were compared to that of vector-transformed eIF2B mutant strains and wild-type strains by streaking and spot assay on synthetic complete (SC) medium containing 2% glucose lacking uracil [27].

### Screening of suppressor protein

eIF2Bβ (*gcd7-201 gcn2∆*) transformants showing Slg^+^ phenotype as that of isogenic wild type were selected and analyzed for Gcd^+^ phenotype by spot assay on SC-medium supplemented with 30 mM 3-AT (3-amino triazole). Transformants showing Slg^+^ and Gcd^+^ phenotype were further screened by spot assay of 10-fold serially diluted culture and by streaking.

Plasmid DNA from the potential *gcd7-201 gcn2∆* transformants (Slg^+^, Gcd^+^) were isolated [28], and *gcd7-201 gcn2∆* mutant *S. cerevisiae* strain were transformed with the rescued plasmid. Simultaneously, the rescued plasmid was sequenced on both the strands at Eurofins Bangalore, (http://www.eurofins.in/) by using YEp24 vector specific primers (S7).

### Functional characterization of suppressor protein

*EMC4* gene from rescued plasmid was amplified using gene-specific primers (Table S3) followed by sub-cloning into pEG(KG) yeast expression vector (containing a *GAL1* promoter and a protease cleavable N-terminal GST tag) at *XbaI/SalI* restriction sites. Gal promoter is repressed by raffinose and induced by galactose.

DH5α was transformed with recombinant plasmids (100 ng) by heat shock method [24]. Rescued plasmid DNA from transformants was sequenced at Eurofins Bangalore, (http://www.eurofins.in/). An error free nucleotide sequence of *EMC4* DNA was obtained. pEG(KG)/*EMC4* plasmids were transformed into *gcd7-201 gcn2∆* strain by LiAc method in order to confirm the Slg^+^ and Gcd^+^ phenotype. The transformation mix was plated on SC medium supplemented with uracil and 2% galactose. *gcd7-201 gcn2∆* and *GCD7 gcn2∆* transformed with pEG(KG) vector alone were used as control.

Plasmid DNA isolation from the recombinant clones was done as described [28] and was transformed again in *gcd7-201* gcn2∆. Spot assay of pEG(KG)/*EMC4* transformants was performed in order to confirm the Slg^+^ and Gcd^+^ phenotype. GST-*EMC4-*based suppression was also confirmed by eviction of pEG(KG) a uracil-based plasmid containing GST-*EMC4* on 5-fluoroorotic acid (FOA) containing medium. 5-Flouroorotic acid (5-FOA) is converted to a toxic product (5-floorouracil) by URA3 gene product. Thus, *S. cerevisiae* cells containing URA3 marker cannot grow on medium containing 5-FOA but are able to grow on medium lacking uracil. Thus, FOA is used to select for the loss of vectors carrying the wild-type URA marker [29]. Colonies from FOA plate were picked and streaked on SC medium without uracil and supplemented with 2% galactose. Plates were incubated at 30 °C for 2 days and were observed for growth phenotype.

### Western blot analysis

The whole cell extract of *gcd7-201 gcn2∆* harboring either pEG(KG) or pEG(KG)/*EMC4* was prepared by glass bead lysis method using Fast Prep (MP Biomedicals). *gcd7-201 gcn2∆* harboring either pEG(KG) or pEG(KG)/*EMC4* were incubated in SC-medium (5 ml) supplemented with 2% raffinose (w/v) at 30 °C for 18 h. Ten milliliters of SC medium supplemented with 2% raffinose (w/v) was inoculated with 1% of overnight grown primary culture followed by incubation at 30 °C. At an absorbance of A _600_ of ~ 0.5, an aliquot was collected as the uninduced control, and the remaining culture was induced by 2% galactose (w/v). Both induced and uninduced cultures were incubated for an additional 3 h at 30 °C.

After incubation, cells were harvested by centrifugation (6000 rpm for 10 min). Protein extraction of both induced and uninduced culture was carried using 20% tri-chloroacetic acid (TCA), and 20 μg of extracted proteins were resolved on SDS–PAGE followed by the transfer to the nitrocellulose membrane (Millipore, Immobilon P 0.45 μm) by electroblotting. The blot was incubated at 4 °C for 1 h in blocking solution containing 5% non-fat dried milk. After incubation, membrane was further incubated with anti-GST antibodies (1:5000, Abcam) overnight at 4 °C. Immunoreactive proteins were detected by using anti-rabbit IgG conjugated to horseradish peroxidase (1:10,000, Abcam) for 1 h. Blots were washed by using PBST (phosphate buffer saline containing TritonX-100) buffer. Finally, the blots were developed using enhanced chemiluminescence kit (ECL, Bio-Rad, Inc. USA).

### Expression of Emc4p in eIF2Bγ, eIF2Bδ, eIF2ε, and *GCN2* mutant *S. cerevisiae* strains

Suppression analysis by Emc4p in other eIF2B mutants was done. eIF2Bγ (H70), eIF2Bδ (H750), eIF2Bε (H1792), and *GCN2* (H591) mutants (Table S1) were transformed with pEG(KG) or pEG(KG)/Emc4. All the transformants were plated on SC-medium lacking uracil supplemented with 2% galactose, and the plates were incubated for 2 days at 30 °C. The transformants were selected and analyzed for Slg^+^ and Gcd^+^ phenotype by streaking and spot assay on SC-medium lacking uracil and containing 2% galactose or SC-medium lacking uracil and containing 2% galactose and 30 mM 3-AT respectively. Plates were incubated for 2 days at 30 °C. eIF2Bγ (H70) a Ts^−^ mutant was also checked for suppression of temperature sensitive (Ts^+^) phenotype by Emc4p at 37 °C.

### Effect of Emc4 protein overexpression on H_2_O_2_-, ethanol-, and caffeine-mediated cell death of eIF2B mutant and wild type *S. cerevisiae* strains

Three different sets of experiments (quantitative assay, spot assay, and halo assay) were performed. Wild-type *GCD7 GCN2*, *GCD7 gcn2∆*, and mutant *gcd7-201 gcn2∆* strains of *S. cerevisiae* containing pEG(KG) or pEG(KG)/Emc4 were incubated for 16 h at 30 °C with shaking in the SC-medium supplemented with either 2% galactose or raffinose (lacking uracil). SC-medium also contained 4 mM H_2_O_2_ [30], 10% ethanol [31], 20 mM caffeine [32], 1.6% DMSO [23], 35 mM Dithiothreitol (DTT) [33], and 1 M NaCl [34] separately. After 16 h of growth, cell density was measured at A _600_ nm using a UV visible spectrophotometer. Spot and halo assays were performed as given by [35, 36].

Ten-fold serially diluted cultures were spotted to check Slg and Gcd phenotypes on SC-medium supplemented with either 2% galactose or raffinose (lacking uracil). The medium was also supplemented 30 mM 3-AT for Gcd^-^ phenotype, 4 mM H_2_O_2_, 10% ethanol, 20 mM caffeine, 1.6% DMSO, 35 mM Dithiothreitol (DTT), and 1 M NaCl. The plates were incubated at 30 °C for 2 days and observed for pattern of yeast cell growth.

For halo assay, filter disks containing 4 mM H_2_O_2_, 10% ethanol, 20 mM caffeine, 1.6% DMSO, 35 mM Dithiothreitol (DTT), and 1 M NaCl were placed on SC agar medium supplemented with either 2% galactose or raffinose (lacking uracil) with uniformly spread culture of *S. cerevisiae* mutant and wild-type strains containing pEG(KG) or pEG(KG)/Emc4. Plates were incubated at 30 °C for 2 days and observed for zone of inhibition. The *S.cerevisiae* strains *GCD7 GCN2*, *GCD7 gcn2∆*, and *gcd7-201 gcn2∆* (Supplementary Table 1) were streaked on YPD plates with or without H_2_O_2_, ethanol, caffeine, DMSO, Dithiothreitol (DTT), and NaCl.

## Results

### Screening of genomic DNA library clones for rescuing slow growth phenotype of *S. cerevisiae* eIF2B mutant strains

Approximately, 30 transformants (*gcd7-201 gcn2∆* transformants) were observed, showing colony size equivalent to that of isogenic wild-type *GCD7 gcn2∆* transformed with vector alone were screened further for Gcd^+^ phenotype. Out of 30 transformants, only the transformant named as Sup-I restored the growth of *gcd7-201 gcn2∆* as well as Gcd^−^ phenotype. The growth of Sup-I clone was very similar to that of isogenic wild-type *GCD7 gcn2∆* transformed with empty vector (Figure S1 a and b).

Plasmid DNA was rescued from Sup-I clone (Figure S1 c) and transformed into *gcd7-201 gcn2∆* strain (Figure S1 d). The growth phenotypes (Slg^+^ and Gcd^+^) of transformants were checked further by streaking mutant transformants (*gcd7-201 gcn2∆*) along with isogenic wild-type strain (Figure S1 d). Results revealed that *gcd7-201 gcn2∆* transformants were of uniform size and showed Slg^+^ and Gcd^+^ phenotype (S1 d and e). This data clearly suggests that a genomic clone (Sup-I) suppressed the Slg^−^ and Gcd^−^ phenotype of *gcd7-201 gcn2∆* mutant strain.

**Fig. 1:**
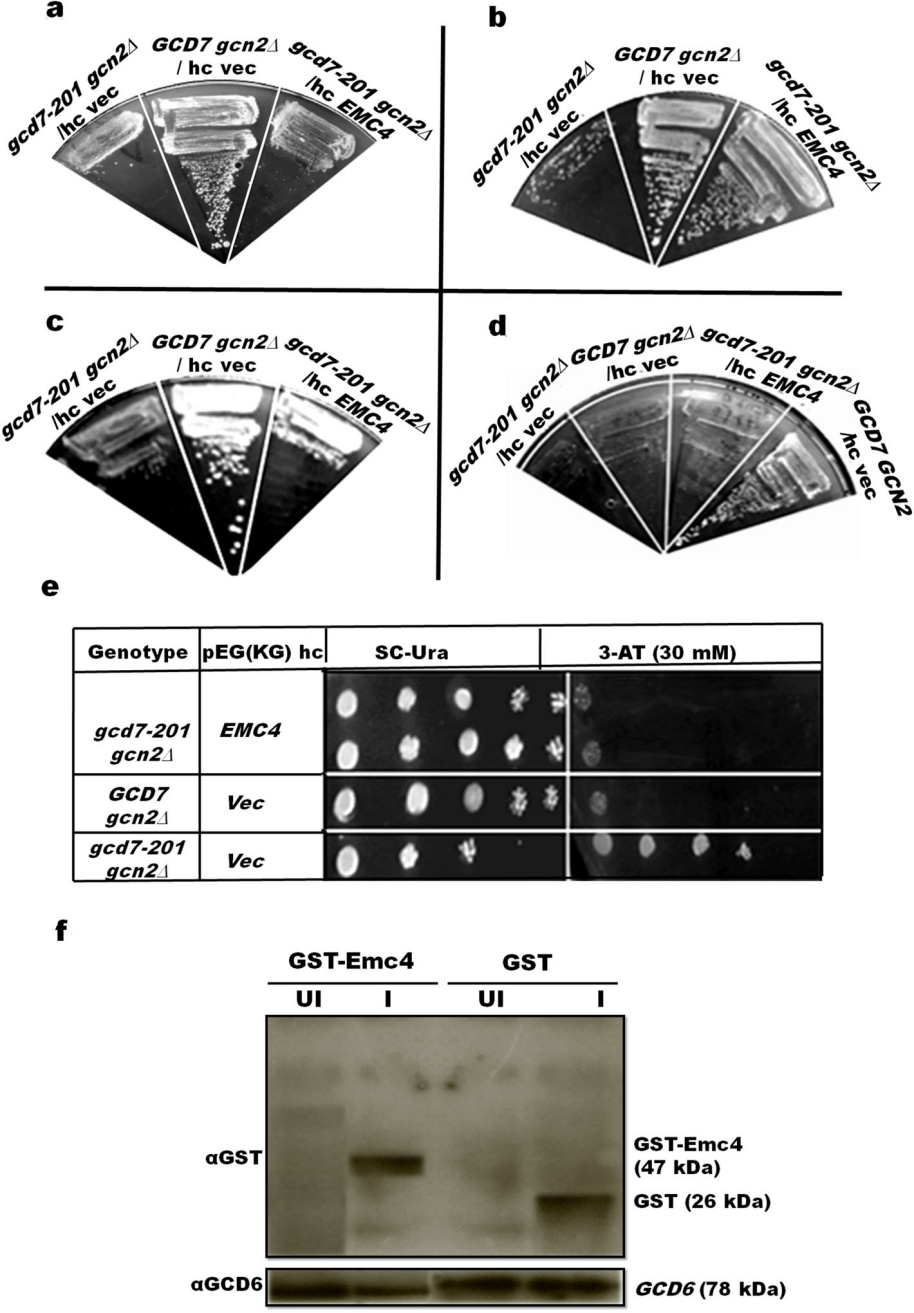
*S. cerevisiae gcd7-201 gcn2∆* mutant strain confers Slg^+^ and Gcd^+^ phenotype when transformed with high copy (hc) pEG(KG)/*EMC4* plasmid. *GCD7 gcn2∆*, *gcd7-201 gcn2∆* harboring hc/*EMC4*, or empty hc vector pEG(KG) were streaked in parallel on SC medium lacking uracil, but either containing (**a**) raffinose or (**b**) galactose. Uracil-based plasmid hc/*EMC4* was evicted on SC medium containing (**c**) FOA, and were further streaked on (**d**) SC-Ura medium. (**e**) Spotting of *GCD7 gcn2∆*, *gcd7-201 gcn2∆* harboring hc/*EMC4*, or empty hc vector pEG(KG) was done on SC medium containing 2% galactose and lacking uracil or SC medium containing 2% galactose, 30 mM 3-AT, and lacking uracil. Plates were incubated at 30 °C for 2 days. (**f**) Western analysis of GST-Emc4p expression in *gcd7-201 gcn2∆* strain with anti-GST antibody. The whole cell protein extracts (20 μg) were prepared from uninduced and 2% galactose-induced cultures of the strain harboring hc/*EMC4*. Samples were then separated on 10% SDS gel followed by Western blotting using anti-GST for GST-Emc4 and anti-Gcd6 antibodies (Loading control). UI, uninduced; I, induced

The Sup-I clone was sequenced using YEp24 specific primers (Table S7). The Sup-I genomic construct revealed the presence of only two complete ORFs including *EMC4 ~* 573 bp and YGL230C *~* 444 bp genes encoding for chaperone and putative protein respectively, whereas *TAN1* (YGL232W) and SAP4 genes were truncated. This data suggests that Sup-I harbors full length *EMC4* that rescued slow growth and Gcd^−^ phenotype of *gcd7-201 gcn2∆* (Fig. 1a).

### Sub-cloning of potential suppressor gene into yeast expression vector, pEG(KG)

*EMC4* gene was amplified using gene-specific primers (Table S3) containing *XbaI* restriction site in the forward primer and *SalI* in the reverse primer followed by cloning in pEG(KG) vector at respective restriction sites. As expected, PCR product of ~ 0.55 kb was observed on agarose gel (Figure S2 b). *EMC4* gene is present on chromosome VII of *S. cerevisiae* genome (http://www.yeastgenome.org/) (S2 a). pEG(KG) vector of 9.3 kb containing GST tag under *GAL1* promoter was used for sub-cloning of *EMC4* gene (Figure S2 c). *EMC4* gene sequence was verified by sequencing, and error free and complete sequence of 573 bp was obtained.

### GST-Emc4 expression rescued the Slg^+^ and Gcd^+^ phenotype of *gcd7-201 gcn2∆*

The *gcd7-201 gcn2∆* was transformed with vector pEG(KG) alone or with GST-*EMC4* expression construct, and the transformants were streaked on SC medium without uracil but supplemented with either raffinose (Fig. 1a) or galactose (Fig. 1b). As expected, *gcd7-201 gcn2∆* transformed with vector alone or *EMC4* construct showed slow growth phenotype on SC medium containing raffinose. Interestingly, GST-Emc4 rescued the growth of *gcd7-201 gcn2∆*, when streaked on SC medium containing galactose (Fig. 1b).

To further analyze the results, GST-*EMC4*, a uracil-based plasmid, was evicted on FOA-containing medium and showed original slow growth phenotype *gcd7-201 gcn2∆* (Fig. 1c) but cannot grow on SC medium without uracil supplementation (Fig. 1d). This data clearly suggests that overexpression of Emc4 rescued the Slg^+^ of *gcd7-201* gcn2∆ mutant.

Further, GST-Emc4 was analyzed for rescuing Gcd^+^ phenotype of *gcd7-201 gcn2∆* mutant strain (Fig. 1e). As expected, *gcd7-201 gcn2∆* transformed with vector alone showed slow growth phenotype in SC medium without uracil supplementation as well as Gcd^−^ phenotype on medium containing 3-AT. Gcd^+^ phenotype of *gcd7-201 gcn2∆* was also rescued when GST-Emc4 was expressed. The suppression of Slg^−^ and Gcd^−^ phenotype by overexpression of GST-Emc4 suggests that Emc4 is involved directly or indirectly in eIF2B-mediated translation regulation.

The expression of GST-Emc4 was verified by western blotting. Whole cell extracts (WCE) of *gcd7-201* gcn2∆ mutant expressing GST-Emc4 or GST alone were observed using anti-GST antibodies. Interestingly, a band of 26 kDa of GST protein was detected in extracts expressing GST alone, while a band of ~ 47 kDa protein was detected in WCE of *gcd7-201 gcn2∆* mutant transformed with GST-Emc4 construct. α-*GCD6* antibodies were used as internal loading control (Fig. 1f).

### Strain specific suppression of eIF2B mutations by Emc4p

**Fig. 2:**
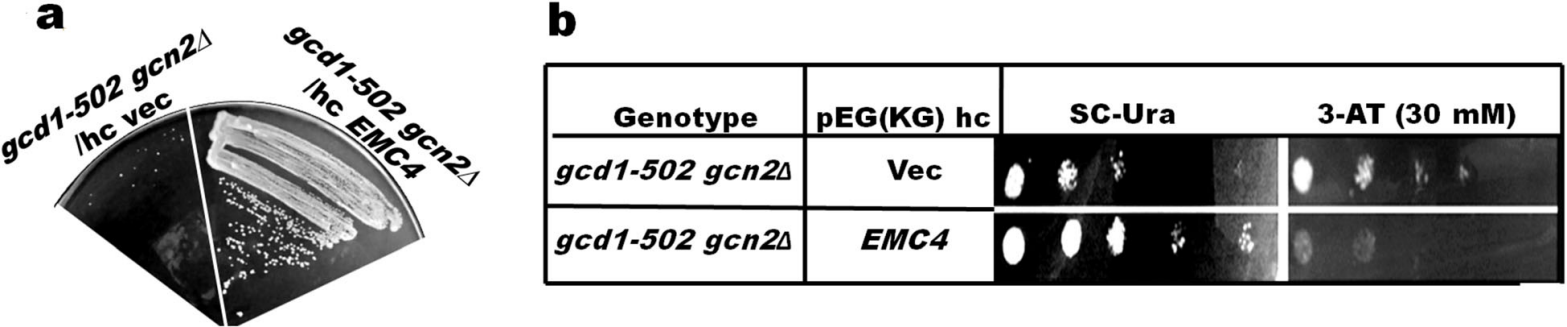
Effect of Emc4p expression on eIF2B mutants. *gcd1-502 gcn2∆* mutant strain rescued Ts^+^, Slg^+^, and Gcd^+^ phenotype when transformed with hc/*EMC4*. (**a**) *gcd1-502 gcn2∆* transformants showing Slg^+^ phenotype at 37 °C were streaked along with *gcd1-502 gcn2∆* transformed with hc vector, pEG (KG). (**b**) Serial dilutions of the transformants showing Slg^+^ phenotype were spotted on SC-Ura and SC medium containing 30 mM-3AT. *gcd1-502 gcn2∆* transformed with hc vector, pEG(KG), was spotted as a control. Plates were incubated for 2 days at 30 °C

Originally, Emc4p was isolated as suppressor of *gcd7-201* mutation of eIF2Bβ. The effect of overexpression of Emc4p on other eIF2B mutations including, *gcd6-1 gcn2∆*, *gcd1-502 gcn2-101*, *gcd12-503 gcn2-101*, and *GCN2* mutant *gcn2::LEU2* (Table S2) was also tested. Interestingly, the Emc4p overexpression rescued temperature sensitive (Ts^−^) phenotype of *gcd1-502* at 37 °C (Fig. 2a), but not that of other eIF2B mutants (data not shown). Emc4p overexpression also rescued Slg^−^ and Gcd^−^ phenotype of *gcd1-502 gcn2-101* (Fig. 2b). These results suggested that Emc4p causes strain-specific suppression of eIF2B mutants.

### GST-Emc4 expression enhances stress tolerance in *S. cerevisiae*

GST-Emc4 overexpression protects wild type and mutant *S. cerevisiae* strains (*gcd7-201 gcn2∆*, *GCD7 gcn2∆*, and *GCD7 GCN2*) against 4 mM H_2_O_2_, 10% ethanol, and 30 mM caffeine-mediated stress. *gcd7-201 gcn2∆*, *GCD7 gcn2∆*, and *GCD7 GCN2* streaked on YPD agar plates showed Slg^−^ phenotype under H_2_O_2_, ethanol, and caffeine stress (Figure S3 a-c). The *S. cerevisiae gcd7-201 gcn2∆*, *GCD7 gcn2∆*, and *GCD7 GCN2* transformed with vector alone showed reduced growth (2-fold) under 4 mM H_2_O_2_, 10% ethanol, and 30 mM caffeine stress in presence of either raffinose or galactose. In contrast, *gcd7-201 gcn2∆*, *GCD7 gcn2∆*, and *GCD7 GCN2* transformed with pEG(KG)/*EMC4* showed normal growth phenotype even under H_2_O_2_, ethanol, and caffeine stress in presence of 2% galactose (Figure S3 a-c ). Emc4p overexpression protects *S. cerevisiae* to H_2_O_2_-, ethanol-, and caffeine-induced cell death. In contrast, no significant protective effect was observed against DMSO, NaCl, and DTT (data not shown).

**Fig. 3:**
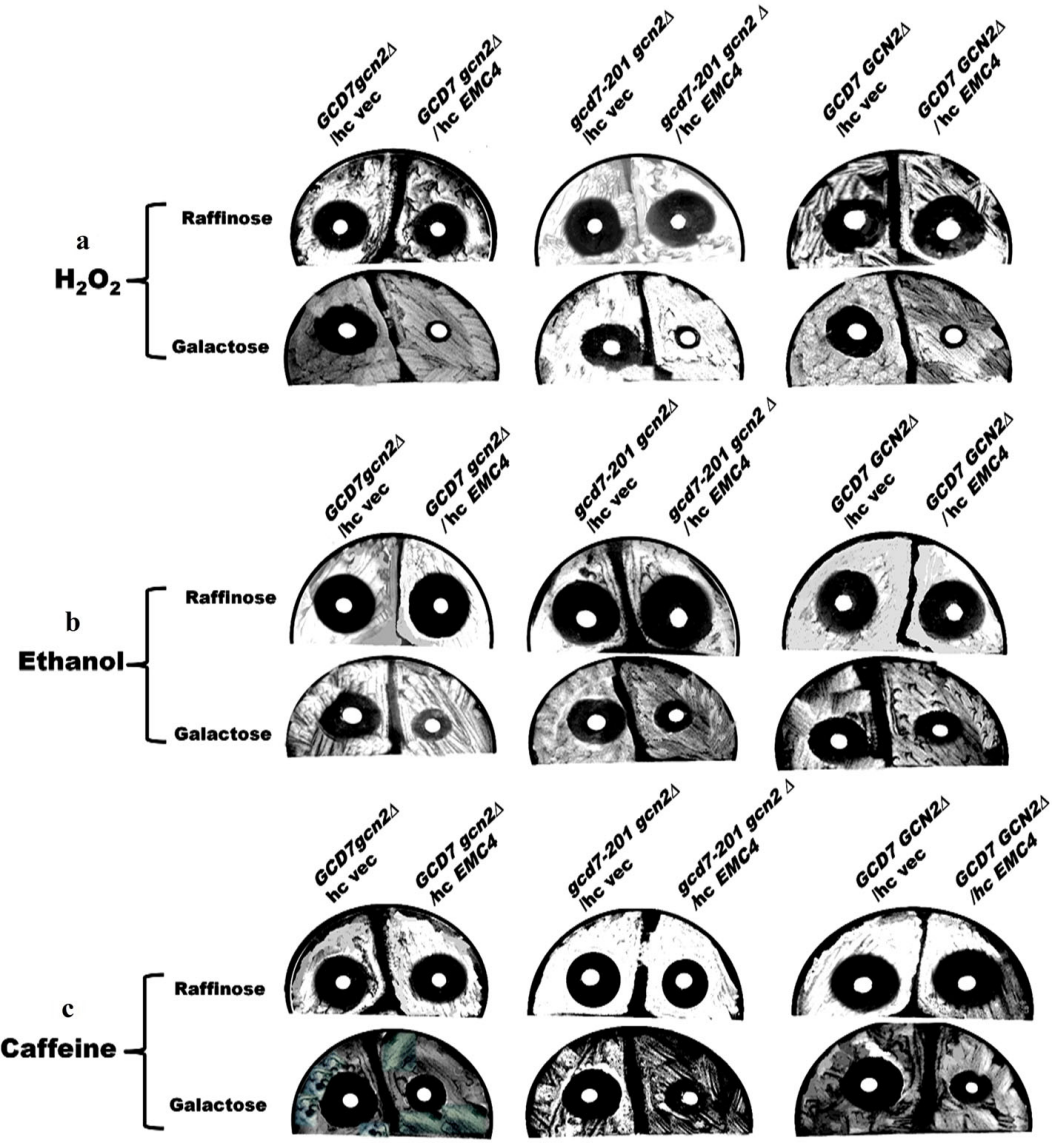
Halo assay to test the effect of H_2_O_2_, ethanol, and caffeine on the growth of eIF2B mutants. Hydrogen peroxide, ethanol, and caffeine halo assays were performed with *gcd7-201 gcn2∆*, *GCD7 gcn2∆*, and *GCD7 GCN2*. *S. cerevisiae* strains harboring either the empty hc vector, pEG (KG), or the hc/*EMC4* gene expressing plasmid were spread on SC medium, containing filter disks soaked in (**a**) H_2_O_2_ (4 mM), (**b)** ethanol (10%), and (**c**) caffeine (30 mM) in the presence of either raffinose or galactose. The plates were incubated at 30 °C for 2–3 days

In spot assays, the *S. cerevisiae* cells *gcd7-201 gcn2∆*, *GCD7 gcn2∆*, and *GCD7 GCN2* overexpressing GST-Emc4 rescued the growth following exposure to H_2_O_2_, caffeine, and ethanol in presence of 2% galactose compared to control cells (Fig. 3a–c).

**Fig. 4:**
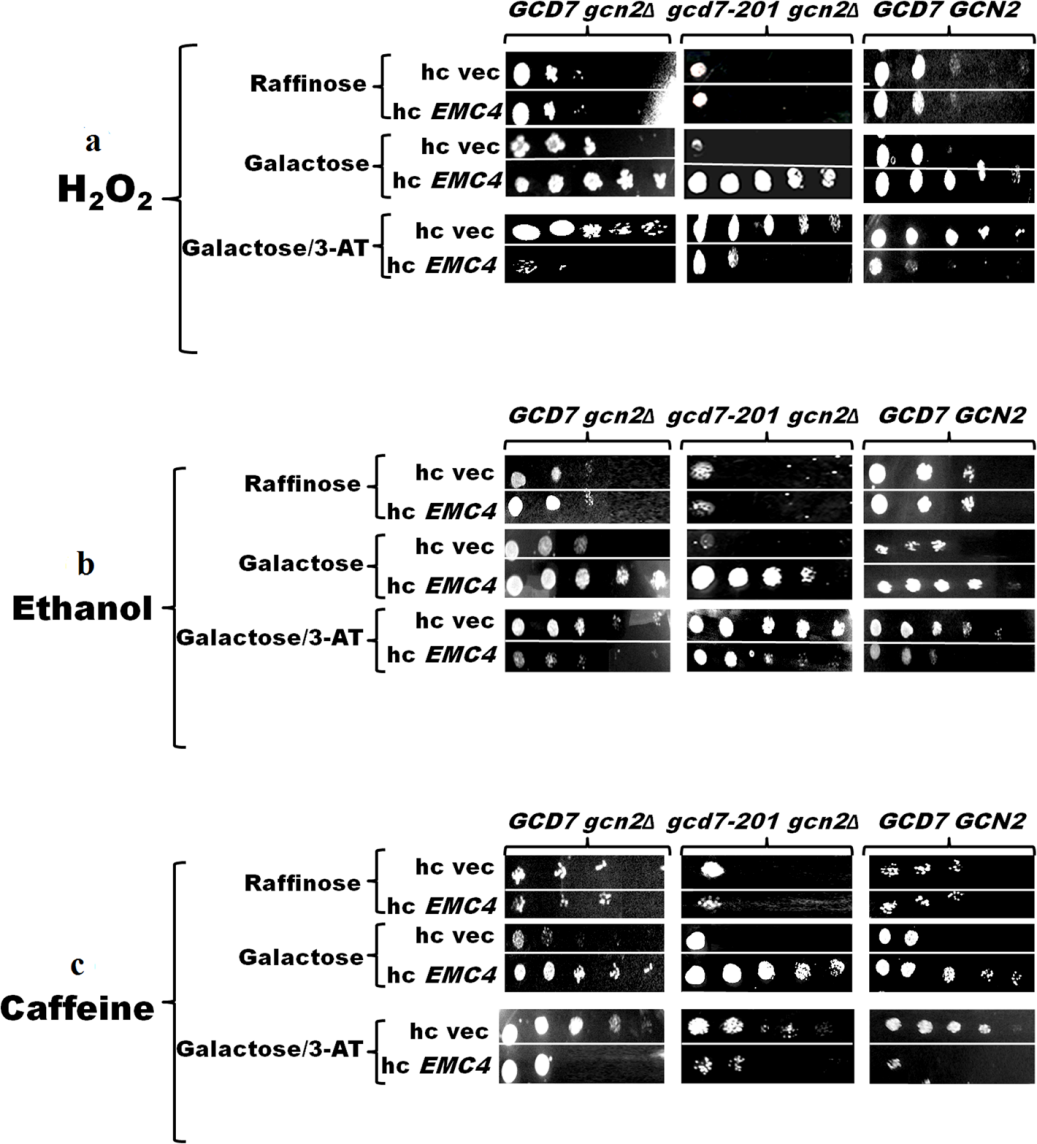
Spot assay to test the effect of H_2_O_2_, ethanol, and caffeine on the growth of eIF2B mutants. Hydrogen peroxide, ethanol, and caffeine spot assays were performed with *gcd7-201 gcn2∆*, *GCD7 gcn2∆*, and *GCD7 GCN2.* Ten-fold serially diluted transformants harboring empty hc, vector pEG (KG), or hc/*EMC4* were spotted on SC-Ura or SC-Ura/3-AT agar plates supplemented with (**a**) H_2_O_2_ (4 mM), (**b**) ethanol (10%), and (**c**) caffeine (30 mM) in the presence of either raffinose or galactose. Cultures were incubated for 2 days at 30 °C

The halo assay reveals that the zone of no growth surrounding the hydrogen peroxide, caffeine, and alcohol containing filter was significantly reduced in *gcd7-201 gcn2∆*, *GCD7 gcn2∆*, and *GCD7 GCN2* strains overexpressing GST-Emc4 in presence of 2% galactose as compared to control cells (Fig. 4a–c). This study strongly suggests that overexpression of Emc4p is capable of preventing cell death caused by high concentrations of H_2_O_2_, alcohol, and caffeine. Moreover, overexpression of Emc4p repairs *gcd7-201 gcn2∆-*based defect in translation initiation.

Based upon the results, we proposed a schematic model (Figure S4) describing the effect of overexpression of Emc4p on eIF2B mutations. During amino acid starvation, H_2_O_2_, ethanol, and caffeine stress in *S. cerevisiae* (wild type), Gcn4p is derepressed. eIF2B mutations are known to derepress the Gcn4p independent of Gcn2p [53]. According to the present model, Emc4p (chaperone protein) overexpression might stabilize the unstable eIF2B complex by properly folding the misfolded subunits of eIF2B complex due to its chaperone activity, which results into stable eIF2-eIF2B interaction and initiates eIF2B GEF activity. This model also describes the role of Emc4p in stress response, where Emc4p overexpression mediates expression of stress response genes under amino acid starvation, H_2_O_2_, ethanol, and caffeine stress.

## Discussion

Eukaryotic initiation factor 2B (eIF2B) is involved in translation initiation/regulation in eukaryotes. Mutations in eIF2B genes lead to deregulation of translation initiation and regulation, causing vanishing white matter disease (VWM). The goal of this study was to identify the extragenic suppressors of *S. cerevisiae* eIF2B mutations corresponding to human eIF2B mutations and to study the role of suppressor protein in eIF2B-mediated regulatory pathways under different stress conditions. *S. cerevisiae* genomic DNA library was constructed and transformed into the eIF2B mutant strains for identification of extragenic suppressors of eIF2B mutations.

Emc4p was observed to suppress the growth defect of *S. cerevisiae*, caused by *gcd7-201 gcn2∆* and *gcd1-502 gcn2-101* mutations. *gcd7-201* is a missense eIF2Bβ^V341D^ mutation corresponding to human eIF2Bβ^V316D^ causing improper folding of eIF2Bβ subunit, as a result eIF2Bδ is excluded from unstable eIF2Bβ^V341D^ complexes, and eventually, rate of protein synthesis is also reduced [18]. Similar results have been reported in previous studies showing that β subunit Gcd7p provides a platform for interaction with Gcd2p helping proper eIF2B complex formation [14]. *gcd1-502* is also a missense mutation of eIF2γ (L480Q). This mutation affects the γ subunit of eIF2B and lowers its GDP dissociation factor (GDF) activity and translation [13].

Endoplasmic reticulum membrane complex subunit 4 *(EMC4)* is a member of endoplasmic reticulum transmembrane complex (EMC complex) and is characterized as a chaperone protein (null mutants are known to induce the unfolded protein response) [5, 6]. This protein also plays an important role in biosynthesis of ionotropic acetylcholine receptors and its inactivation reduces the total number of nicotinic acetylcholine receptors (AChRs) present on plasma membrane of muscle and neuronal cells [33]. Thus, Emc4p is important for the development of muscle and neuronal cells. Slight change in the levels of Emc4p can cause brain-related disorders like vanishing white matter disease. But no relevance of Emc4p and VWM has been reported till now. Studies in mammalian systems have reported the high levels of EMC4 protein in the brain and specially during developmental stages of the organism (http://www.proteinatlas.org). Emc4p is also involved in stress response as described in a study which shows that overexpression of YGL230C inhibits the hydrogen peroxide-mediated oxidative stress and cell death in *S. cerevisiae* [30]. EMC4 is also involved in tethering of endoplasmic reticulum with mitochondria, important step for phospholipid metabolism. Lipids being important components of neuronal cells require more lipids, and defect in lipid metabolism in nerve cells can cause neurodegenerative disease [37]. These studies support our data that Emc4p is an important protein, regulating translation in some or other way in brain cells.

Emc4p is also involved in hydrogen peroxide-mediated oxidative stress response in S. cerevisiae [30]. Considering this fact, *gcd7-201 gcn2∆*, *GCD7 gcn2∆*, and *GCD7 GCN2* strains of *S. cerevisiae* overexpressing Emc4p were also tested for resistance to other translation-inhibiting compounds (H_2_O_2_, ethanol, caffeine, DMSO, DTT, and NaCl). Surprisingly, Emc4p overexpression rescued both the Slg^−^ and Gcd^−^ phenotype of *GCD7 GCN2*, *GCD7* gcn2∆, and *gcd7-201 gcn2∆ S. cerevisiae* strains under H_2_O_2_, ethanol, and caffeine stress but no effect was observed for DMSO-, DTT-, and NaCl-mediated stress conditions. Here we demonstrated that the overexpression of Emc4p suppressed the H_2_O_2_-, ethanol-, and caffeine-mediated growth inhibition of *gcd7-201 gcn2∆* mutant and wild-type strains in Gcn2p independent manner possibly by modulating/targeting the eIF2B for translational regulation. According to the present study, H_2_O_2_, ethanol, and caffeine might be used as chemical modulators to study eIF2B-mediated pathways leading to stress responses as described previously [16]. Earlier study has proposed that Tan1p overexpression confers resistance to *GCD7 GCN2*, *gcd7-201 gcn2∆*, and *GCD7 gcn2∆* growth defect under ethanol, H_2_O_2_, and caffeine stress [38].

Our studies are consistent with the previous reports of Gcn2 independent Gcn4 induction and translation regulation. For illustration, role of butanol mediated induction of *GCN4* by a Gcn2p-independent manner has already been reported [39]. Oxidative stress (H_2_O_2_) causes translation inhibition by Gcn2 or eIF2α phosphorylation independent manner [40]. The molecular mechanisms of these processes are not fully understood.

Although, the mechanism by which Emc4p functions in translation regulation is still unknown, this study provides strong evidence that Emc4p protein in some or other way (possibly by stabilizing the eIF2-eIF2B interaction) is involved in the eIF2B-mediated translation initiation and regulation pathway. More importantly, the role of Emc4p in the brain describes and supports our study and could provide a tool for understanding the mechanism behind vanishing white matter disease.

## Conclusions

In this work, we identified Emc4p as an extragenic suppressor of eIF2B mutations (*gcd7-201 gcn2∆* and *gcd-502 gcn2∆*). Our results revealed that Emc4p suppresses the slow growth and general control derepression phenotypes of *S. cerevisiae* eIF2B mutations, corresponding to human eIF2B mutations. Emc4p does this by its chaperone activity in which it properly folds and stabilizes the mutant subunits (*gcd7-201* and *gcd1-502*)*.* This indicates that interaction of these two subunits is important for eIF2B activity. In addition, Emc4p also suppresses the *S. cerevisiae* growth defect under H_2_O_2_, ethanol, and caffeine stress which clearly indicates the role of Emc4p in eIF2B-mediated translation initiation and regulation most importantly in the brain. Our results help in understanding the mechanism behind VWM disease as our results are supported by previous studies in which the different roles of Emc4p in the brain and in stress response are clearly described.

## Supplementary information

**Additional file 1.** Supplementary file

## Data Availability

Data provided as supplementary.

## Abbreviations

VWM: vanishing white matter
eIF2B: Eukaryotic initiation factor 2B
EMC4: ER membrane protein complex subunit 4
Slg: slow growth
Gcd: general control derepression
3-AT: 3-Amino triazole
SC: synthetic complete medium
Cfu: Colony forming units
