# Supplementary material for: Saccharomyces cerevisiae ER membrane protein complex subunit 4 (EMC4) plays a crucial role in eIF2B-mediated translation regulation and survival under stress conditions
Source: J Genet Eng Biotechnol. 2020 Jun 1;18:15. doi: 10.1186/s43141-020-00029-7 (PMC7261713; doi:10.1186/s43141-020-00029-7)
Supplement: Supplementary file 1 — Additional file 1. Supplementary file [file 43141_2020_29_MOESM1_ESM.docx]

**SUPPLEMENARY DOCUMENT**

Supplementary Figure 1


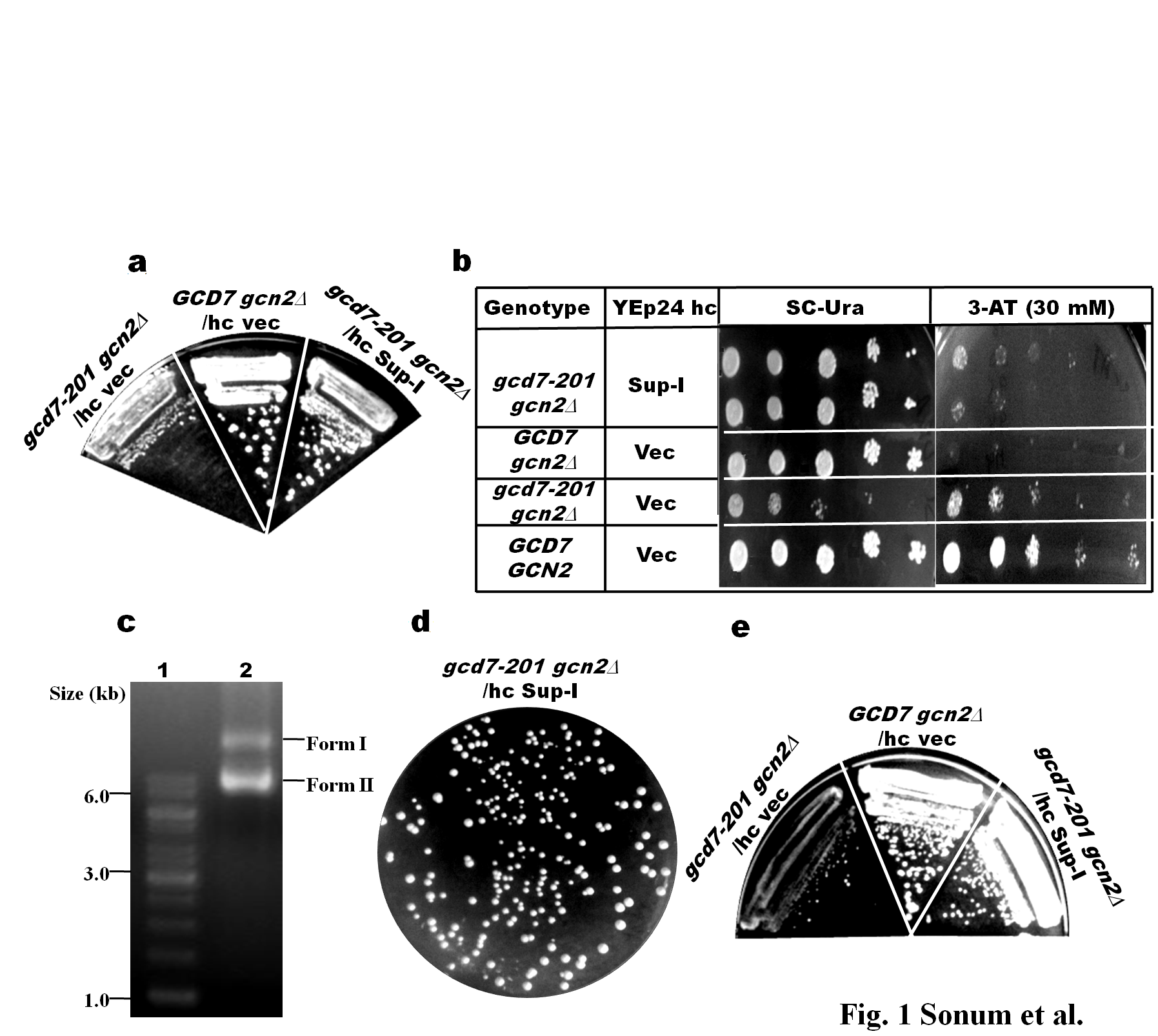


**Supplementary Figure 1.** Screening of *gcd7-201* *gcn2∆* transformants for extragenic suppressors. *gcd7-201* *gcn2∆* mutant strain transformed with *S. cerevisiae* genomic DNA library rescued Slg^+^ and Gcd^+^ phenotype.  **a** *gcd7-201* *gcn2 ∆* transformant showing Slg^+^ phenotype (Sup-I) was streaked along with *gcd7-201* *gcn2∆* and *GCD7* *gcn2∆* transformed with YEp24 high copy (hc) vector. **b** Serial dilutions of the transformants (Sup-I) showing Slg^+^ phenotype, were spotted on SC-Ura and SC medium containing 30 mM-3AT separately. *GCD7* *gcn2∆* and *GCD7 GCN2∆* transformed with vector were spotted as a control. **c** The transformants (Sup-I) showing Slg^+^ and Gcd ^+^ phenotype were used for rescuing plasmid as indicated in lane 2. **d** The rescued plasmid was transformed into *gcd7-201* *gcn2∆* mutant strain and (e) further verified by streaking on SC-Ura medium as indicated.

Supplementary Figure 2


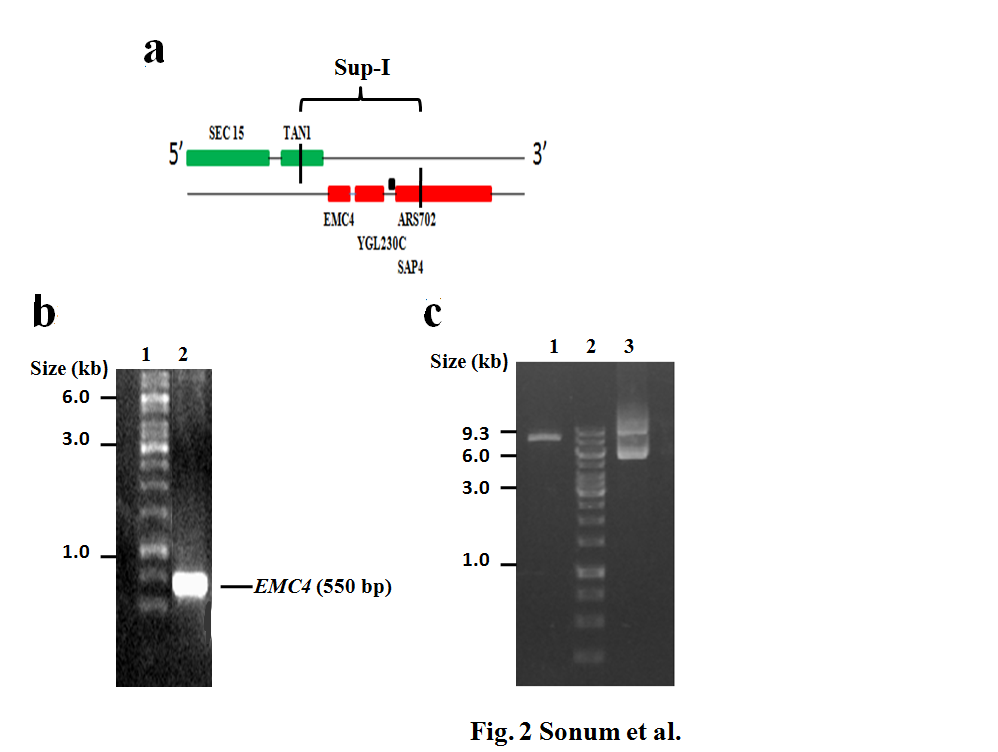


**Supplementary Figure 2.** PCR amplification of *EMC4* gene. **a** Schematic representation of Sup-I genomic construct showing complete ORF of *EMC4*, YGL230C and truncated *TAN1* and *SAP4* on chromosome VII, **b** PCR amplification of the *EMC4* gene from genomic clone (Sup-I) and separated on 1% agarose gel (lane 2) and **c** pEG(KG) plasmid DNA was isolated as indicated (lane 3) and digested with *XbaI* and *SalI* (lane 1). In **b** and **c,** lane 1 and 2 represent a molecular size marker (kb) respectively.

Supplementary Figure 3
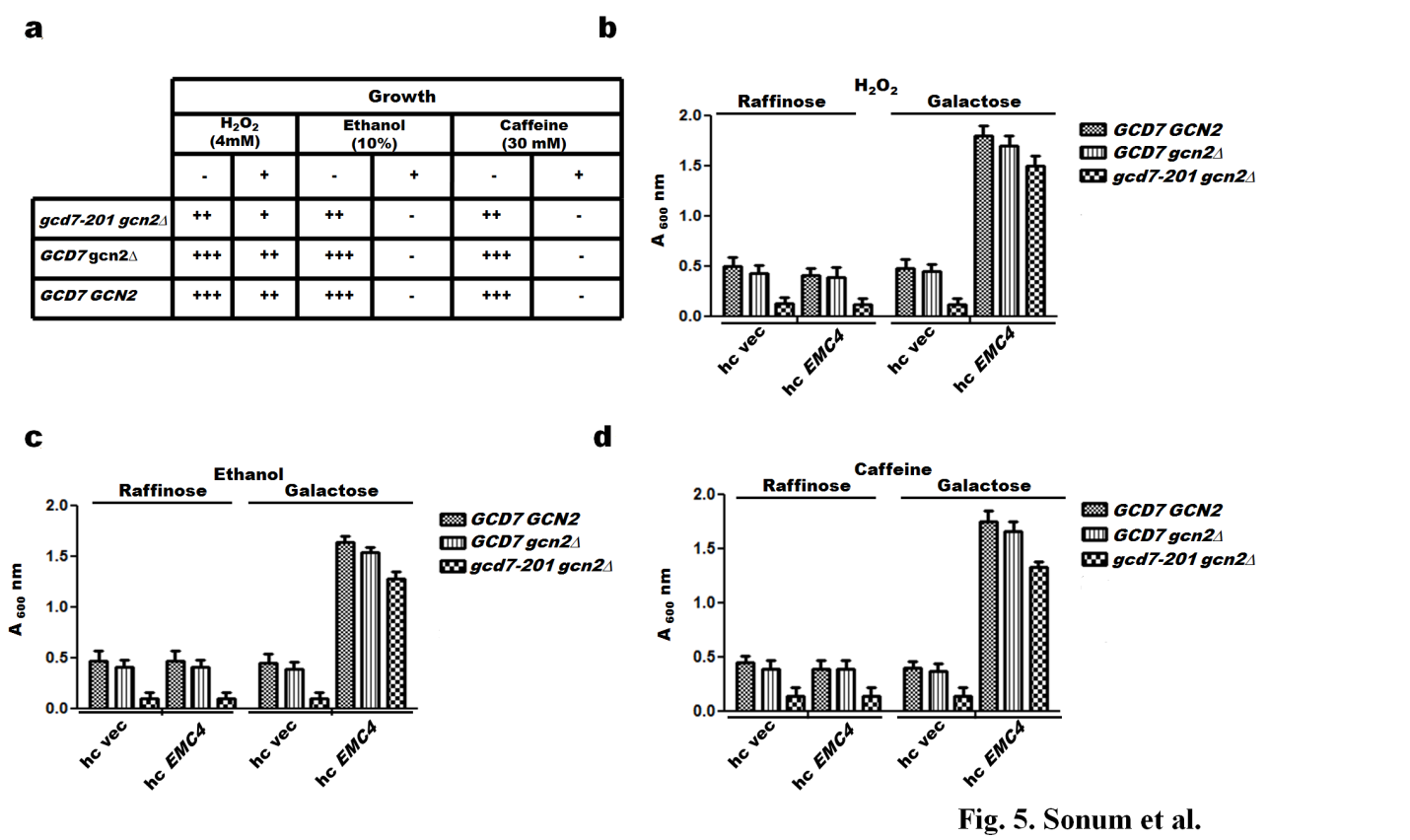


**Supplementary Figure 3.** Effect of H_2_O_2,_ ethanol and caffeine on the growth of *gcd7-201 gcn2∆*, *GCD7 gcn2∆* and *GCD7 GCN2∆* *S.* *cerevisiae* strains***.* a** *gcd7-201 gcn2∆,* *GCD7 gcn2∆* and *GCN2 GCN2∆* *S. cerevisiae* strains were streaked on YPD agar plates containing 4 mM H_2_O_2_, 10% ethanol and 30 mM caffeine. Number of plus signs (+) indicate comparative visible growth. Negative signs (-) indicates no visible growth. *gcd7-201 gcn2∆,* *GCD7 gcn2∆* and *GCN2 GCN2∆* strains containing either hc vector, pEG(KG) or hc/*EMC4* were incubated in SC medium supplemented with **b** 4 mM H_2_O_2_, **c** 10% ethanol and **d** 30 mM caffeine in the presence of raffinose or galactose. Cultures were grown for 16 h at 30ºC. Cultures with raffinose supplementation were served as control. The cell density was observed by measuring absorbance at 600 nm (A _600_). Data of three independent experiments was plotted with standard deviation*.*

Supplementary Figure 4
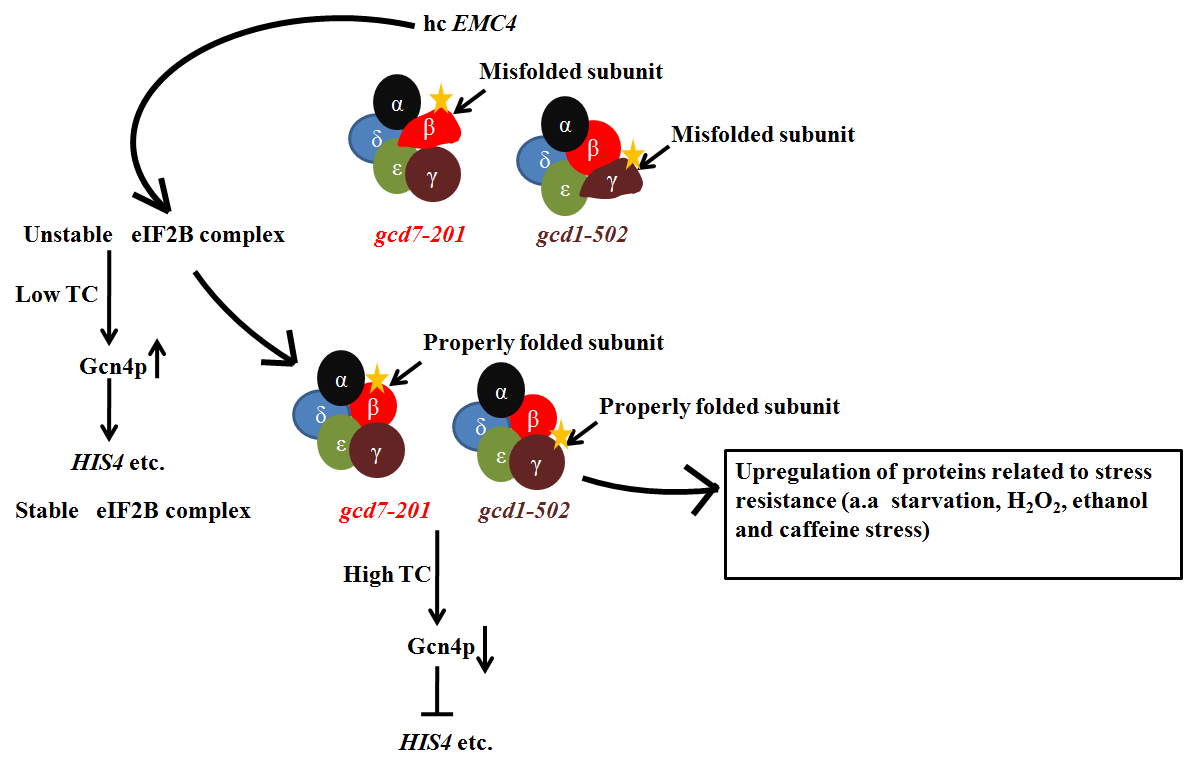


**Supplementary Figure 4.** Overexpression of GST-Emc4p stabilizes the eIF2B complex by modulating the improper folded subunits in eIF2B complex*.* Mutations in *GCD7* and *GCD1* subunit of eIF2B complex destabilize the whole eIF2B complex eventually lowering the TC complex and inducing Gcn4p expression. Interestingly, Emc4p overexpression increases the TC level and represses the Gcn4p expression by stabilizing the eIF2B complex as shown. Similarly, the overexpression of Emc4p also protects the eIF2B mutant strains from H_2_O_2,_ ethanol and caffeine stress *via* eIF2B mediated translation regulation.

Supplementary Table 1

**TABLE 5. Description of *Saccharomyces cerevisiae* strains used in this study**

**Strain Genotype Source or reference**

Alan G. Hinnebusch

H4 *mata leu2-3 leu2-112* *ura3-52*

Alan G. Hinnebusch

H1794 *mat@ leu2-3 leu2-112 ura3-52* *gcd7-201* *gcn2::LEU2*[p500]

Alan G. Hinnebusch

H1795 *mat@ leu2-3 leu2-112 ura3-52* *GCD7* *gcn2::LEU2*[p500]

Alan G. Hinnebusch

H1792 *mat@ ura3-52* gcd6-1 *gcn2::LEU2*[p500]

Alan G. Hinnebusch

Alan G. Hinnebusch

H1793 *mat@ ura3-52* *GCD6* *gcn2::LEU2*[p500]

Alan G. Hinnebusch

H750 *mata* gcn2::*LEU2 leu2-3 leu2-112 ura3-52*

H591 *mat@ gcd12-503 gcn2-101 gcn3-101 his1-29 ura3-52 ino1* (HIS4-lacZ, ura3-52)

H70  *mat@ his1-29* gcn2-101 *gcn3-101* *ura3-52* *gcd1-502* (HIS4-lacZ, ura3-52)

Alan G. Hinnebusch

Supplementary Table 2

**TABLE 6. Nomenclature of *Saccharomyces cerevisiae* strains used in this study**

**Strain Nomenclature used Source or reference**

Alan G. Hinnebusch

H4 *GCD7 GCN2*

Alan G. Hinnebusch

H1794 *gcd7-201* *gcn2∆*

Alan G. Hinnebusch

Alan G. Hinnebusch

H1795 *GCD7gcn2∆*

H1792 *gcd6-1 gcn2∆*

Alan G. Hinnebusch

H1793 *GCD6 gcn2∆*

Alan G. Hinnebusch

Alan G. Hinnebusch

H750 gcn2::*LEU2*

H591 *gcd12-503 gcn2-101*

Alan G. Hinnebusch

H70  *gcd1-502 gcn2-101*

Supplementary Table 3

**TABLE 7. Primers used in this study**

**Name Nucleotide sequence (5´-3´) Source**

This study

YEp24-F TGGAGCCACTATCGACTACG

This study

This study

YEp24-R GGCGATATAGGCGCCAGCAA

EMC-F TAGCTCTAGAATGAGTGAACAGGAG

This study

EMC-R TCTAGTCGACTCAATCGGAAAACCA
